# Brentuximab vedotin as a bridge to combination chemotherapy in gray zone lymphoma with severe liver impairment: a case report

**DOI:** 10.3389/fonc.2023.1254256

**Published:** 2024-01-19

**Authors:** Roshan Asrani, Turgot Bora Cengiz, Bruce E. Petersen, Theodora Anagnostou, Joshua D. Brody

**Affiliations:** ^1^ Department of Hematology/Oncology, Icahn School of Medicine at Mount Sinai, New York, NY, United States; ^2^ Department of Nuclear Medicine, Icahn School of Medicine at Mount Sinai, New York, NY, United States; ^3^ Department of Pathology, Icahn School of Medicine at Mount Sinai, New York, NY, United States

**Keywords:** CD30+ lymphoma, targeted therapy, hyperbilirubinemia, Mdm4, IKBKE

## Abstract

**Background:**

Gray zone lymphoma (GZL) is a rare lymphoma subtype characterized by features intermediate between diffuse large B-cell lymphoma (DLBCL) and classic Hodgkin lymphoma (cHL). The optimal first-line treatment for GZL remains undefined, particularly for patients with poor performance status or baseline organ impairment. Brentuximab vedotin (BV), a targeted therapy that binds to CD30, a TNFR superfamily member involved in NF-kB signaling, has shown promise in the treatment of CD30-positive lymphomas. However, its use in GZL, especially in patients with severe liver impairment, has not been reported previously.

**Case description:**

We present a case of a 37-year-old male with GZL and severe liver impairment at initial presentation. The patient initially received monotherapy with BV, which resulted in a marked improvement in liver enzymes and bilirubin levels. Subsequently, combination cytotoxic chemotherapy consisting of dose-adjusted etoposide, prednisone, cyclophosphamide, and doxorubicin (DA-EP_CH) was added. Repeat imaging revealed near complete resolution of lymphadenopathy and significant reduction in hepatosplenomegaly. The patient completed a full course of chemotherapy and achieved a complete response. Follow-up examinations showed no evidence of recurrent disease, and the patient resumed full-time work.

**Discussion:**

GZL poses diagnostic challenges due to its overlapping features with DLBCL and cHL. Accurate diagnosis relies on comprehensive histopathological evaluation, immunophenotyping, and molecular analysis. The optimal first-line treatment for GZL remains uncertain. BV shows promise as an addition to chemotherapy in GZL, even in the presence of severe liver impairment. The molecular pathogenesis of GZL is complex and heterogeneous, frequently involving aberrant NF-kB signaling and impaired apoptosis regulation via loss of TP53 expression. Understanding the underlying molecular mechanisms is essential for developing targeted therapies and identifying predictive biomarkers for treatment response.

**Conclusion:**

This case demonstrates the successful use of BV as a bridge to cytotoxic chemotherapy in a GZL patient with severe liver impairment, highlighting its potential safety and efficacy even in the setting of end-organ failure. Further investigation is warranted to define optimal treatment strategies, identify predictive biomarkers, and improve outcomes for patients with this rare and challenging lymphoma subtype.

## Highlights

- Gray zone lymphoma (GZL) is a rare lymphoma with features intermediate between diffuse large B-cell lymphoma and classic Hodgkin lymphoma.
- The optimal first line treatment for GZL is not well defined, especially for patients with poor performance status.
- Brentuximab Vedotin (BV) is a promising addition to chemotherapy that targets CD30, a TNFR superfamily member, which drives NF-kB signaling.
- BV can likely be safely used in GZL patients even in the setting of end-organ failure and as a bridge to cytotoxic chemotherapy.
- The molecular pathogenesis of GZL is complex and heterogeneous but frequently includes aberrant NF-kB signaling and impaired apoptosis regulation via loss of TP53 expression.

## Introduction

Gray zone lymphoma (GZL) denotes a previously unclassified group of lymphomas with features intermediate between diffuse-large B-cell Lymphoma (DLBCL) and classic Hodgkin Lymphoma (cHL). Incidence is difficult to characterize due to its rarity, but a population registry in the United States estimated 1.27 cases per million in 2016 (1). Clinically, patients are usually recognized with primary mediastinal disease, however, multi-center cohorts have reported non-mediastinal cases as well (2). Since the neoplasm shares features of both DLBCL and cHL, accurate diagnosis of GZL remains complex despite adequate tissue typing by immunohistochemistry (IHC) and advances in molecular diagnostics. Often, final diagnosis of GZL is only elucidated upon repeat biopsy as tumor samples can be heterogeneous (2 ).Defining features include morphology with an abundance of tumor cells organized in sheets, with nuclear pleomorphism, and with a paucity of background inflammatory eosinophils and histiocytes (3). Immunohistochemistry findings likewise show features of DLBCL and cHL with most cases exhibiting positivity for CD20 along with CD30 and MUM1. In contrast to cHL, CD15 expression is usually negative and rare cases exhibit EBV positivity.

Optimal therapeutic approaches for initial treatment of GZL remain controversial, with limited available prospective data. Current practice favors the use of DLBCL based chemoimmunotherapy regimens such as R-CHOP (rituximab, cyclophosphamide, doxorubicin hydrochloride, vincristine sulfate, and prednisone) or DA-EPOCH-R (dose-adjusted etoposide, prednisone, vincristine, cyclophosphamide, doxorubicin, rituximab) as opposed to cHL directed therapy with ABVD (adriamycin, bleomycin sulfate, vinblastine sulfate, and dacarbazine) +/- the addition of rituximab (4). In a retrospective series, the 2-year progression free survival (PFS) was inferior in patients receiving cHL based therapy with ABVD +/- R vs DLBCL regimens (CHOP +/- R, DA-EPOCH +/- R) at 23% vs 52% respectively, acknowledging difficulties in accounting for potential confounding factors such as differences in rituximab use (91% in CHOP vs 18% in ABVD) (2). Additionally, there is limited guidance regarding the management of patients who are intolerant to chemotherapy on initial evaluation due to poor fitness, advanced age, or end-organ impairment. One case report has described brentuximab vedotin (BV) + CHP (cyclophosphamide, doxorubicin, and prednisone) + R as an effective first line regimen for the treatment of GZL (5). BV in combination with R-CHP has also previously been found to be safe and efficacious in CD30+ B-cell lymphomas, with an overall response rate (ORR) of 100%, and 86% of patients achieving complete remission (CR) (6).

Currently, there has been no reported use of BV in GZL patients with end-organ impairment preventing administration of chemotherapy. BV monotherapy has been reported as a successful bridge to cytotoxic chemotherapy in relapsed/refractory (R/R) cHL with severe liver impairment (7), despite the manufacturer’s label recommendation to “avoid use” in severe impairment (Child Pugh B or C) (8).

Herein, we report the first case of the successful use of BV as a bridge to combination cytotoxic chemotherapy in a patient with GZL and severe liver impairment at initial presentation.

## Clinical case

**Figure 1:**
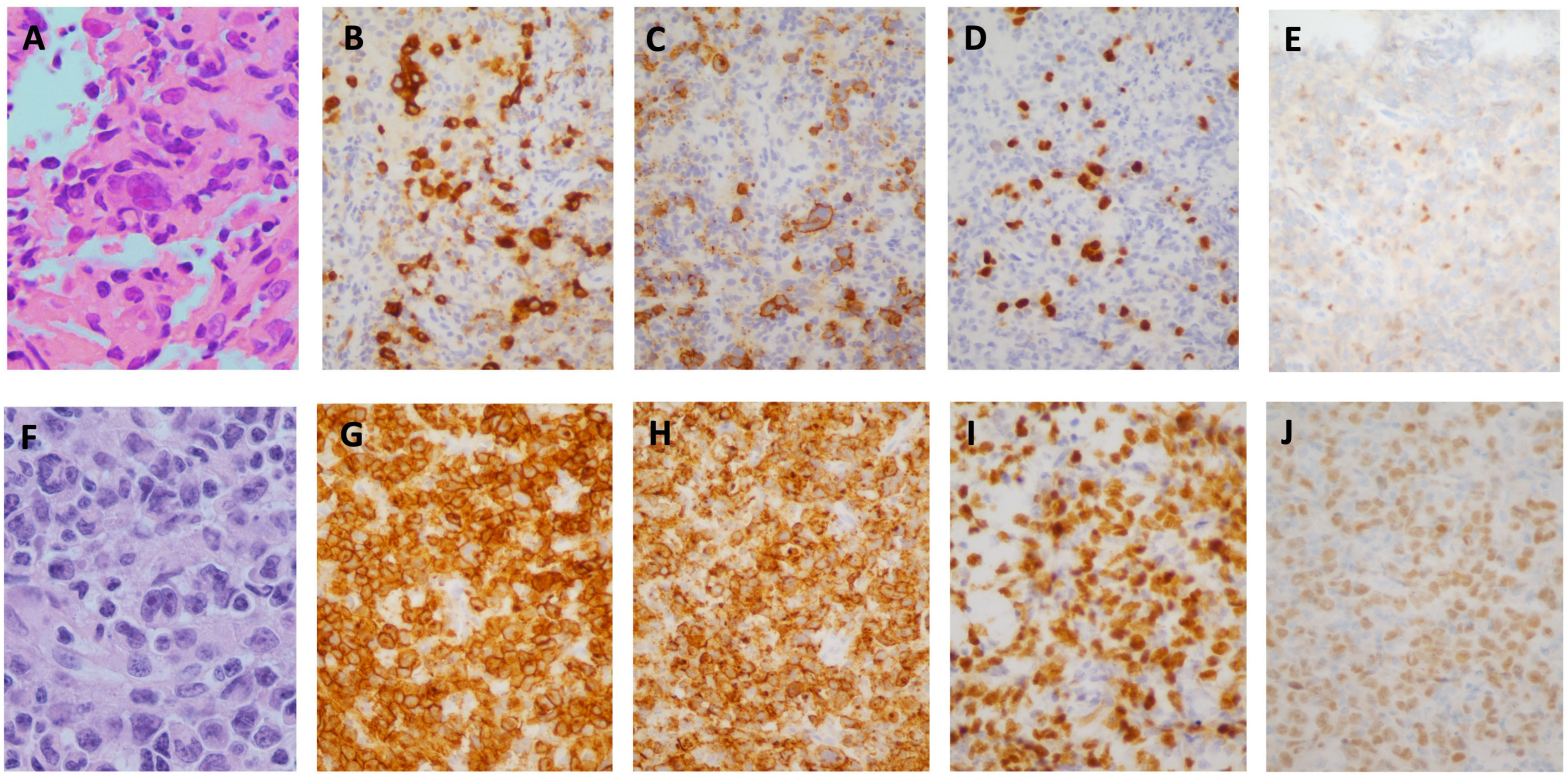
Case Pathology. **(A–E)** H&E stain of initial core biopsy of left cervical lymph node showing atypical lymphocytic infiltrate consisting of isolated large, pleomorphic, atypical lymphoid cells, including cells with features of Hodgkin/Reed-Sternberg cells **(A)**, positive for CD30 **(B)**, CD15 **(C)**, strongly positive for PAX5 **(D)**; with equivocal weak labeling for OCT2 **(E)**. **(F, J)** H&E stain of core biopsy of subsequent left axillary lymph node showing similar-appearing atypical lymphoid cells in confluent sheets **(F)**, positive for CD30 **(G)**, CD15 **(H)**, PAX5 **(I)**, OCT2 **(J)**. (**A, F**, X1000; **B–E, H–J** X400).

A 37 year-old male presented to his primary care physician with new right cervical lymphadenopathy, progressing over 3 months, as well as drenching night sweats and low-grade fevers. Computerized Tomography (CT) scan of the neck with contrast revealed numerous non-enhancing, non-necrotic, enlarged bilateral level II and III lymph nodes measuring 2cm each and a conglomerate matted lymph node, measuring 3.3 anterior posterior (AP) x 6.4 cm transverse, in the left supraclavicular fossa. A level III ultrasound-guided cervical lymph node core-needle biopsy showed a few large mononuclear atypical lymphoid cells with prominent nucleoli in the background of small lymphocytes and histiocytes. A few Reed-Sternberg-like cells were also present. By immunohistochemistry, the neoplastic cells were CD30+, CD15+, PAX5+, MUM1+, BCL6+, CD3-, CD5-, BCL2-, with a few cells positive for CD20, suggesting a differential diagnosis including cHL versus T-cell/ histiocyte-rich large B-cell Lymphoma (**Figure 1**).

A subsequent left axillary lymph node core-needle biopsy showed sheets of large, atypical, pleomorphic lymphoid cells with prominent nucleoli; CD30+, CD15+, CD45-, PAX5+, OCT2+, BOB1+, MUM1+, CD20-, CD3-, CD5- by immunohistochemical staining (**Figure 1**). Flow cytometry was unrevealing. Cytogenetics and FISH studies were significant for tetraploidy of MYC (8q24.21) and BCL2 (18q21) as well as 3-5 copies of IGH (14q32). The absence of CD45 and CD20 expression and presence of CD15 expression were inconsistent with CD30+ DLBCL. Morphological and immunophenotypic features intermediate between those of cHL and DLBCL led to the diagnosis of GZL. Next generation sequencing was performed on the biopsy specimen and showed low tumor mutational burden (5 muts/Mb), microsatellite stability (MSS), amplification of IKBKE (11 copies) and MDM4 (11 copies) and mutation of ASXL1 (D740fs*6) with variant allele frequency (VAF) of 13.21%.

The patient developed jaundice, and was admitted to the hospital, where laboratory findings were significant for White blood cell count (WBC) 0.8 x10^3^/uL, Hemoglobin (Hb) 6 g/dL, Platelets 23,000/uL, Lactate Dehydrogenase (LDH) 942 U/L, Erythrocyte sedimentation rate (ESR) 40 mm/h, C-reactive protein (CRP) 15 mg/L, total bilirubin 5 mg/dL, Aspartate transaminase (AST) 86 units/L, Alanine transaminase (ALT) 64 units/L, albumin 2.5 g/dL, and uric acid 3.5 mg/dL. Staging evaluation was performed with positron emission tomography–computed tomography (PET-CT), which revealed numerous enlarged hypermetabolic cervical chain lymph nodes bilaterally with a peak SUV of 14.3, an enlarged left axillary lymph node of 2.9cm with SUV of 15.5 and bilateral retroperitoneal lymphadenopathy. Splenomegaly with enlargement to 35cm was noted with hypermetabolism (SUV 11), as well as hepatomegaly (23.4 cm) with heterogenous FDG uptake throughout the parenchyma. Symmetric radiotracer activity was noted in axial and proximal appendicular bone marrow. A bone marrow biopsy was positive for involvement with lymphoma. Based on these findings the patient was classified as stage IVA. Echocardiography revealed an ejection fraction of 60% with no valvular disease.

**Figure 2:**
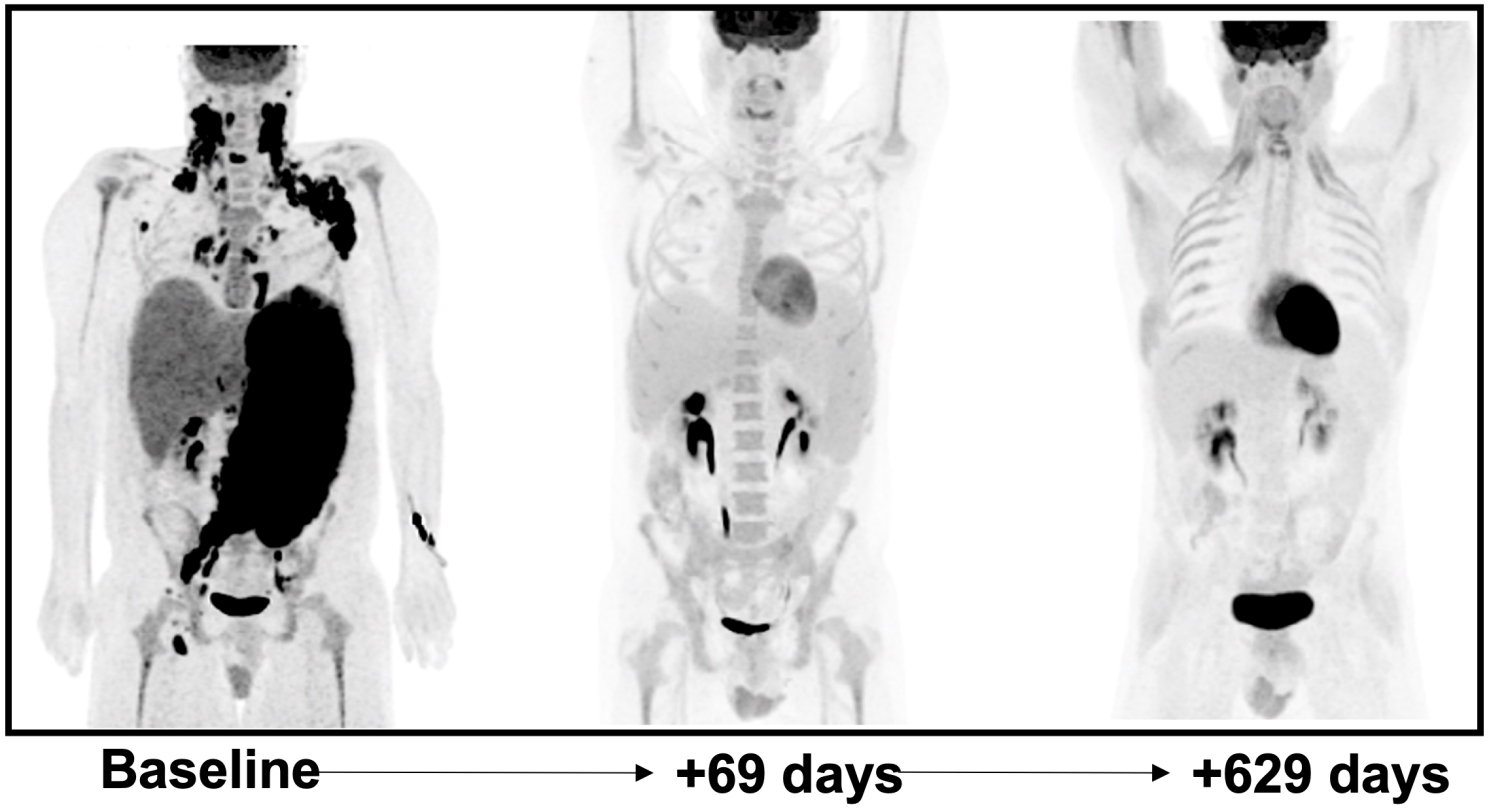
Coronal maximum-intensity-projection (MIP) images of 18F-FDG PET-CT at baseline post-treatment initiation interval at 69 days later and surveillance follow-up 629 days later. At +69 days post-treatment initiation there is significant decrease in splenomegaly (33.7 cm to 21.7 cm) and hepatomegaly (23.1 cm to 20.8 cm) with interval resolution of bulky FDG avid lymphadenopathy in the bilateral cervical and left axillary regions. The patient was treated with G-CSF prior to the 2nd panel image which likely explains diffuse osseous uptake which was noted. Follow-up examination >1.5 years later continues to show no evidence of disease.

**Figure 3:**
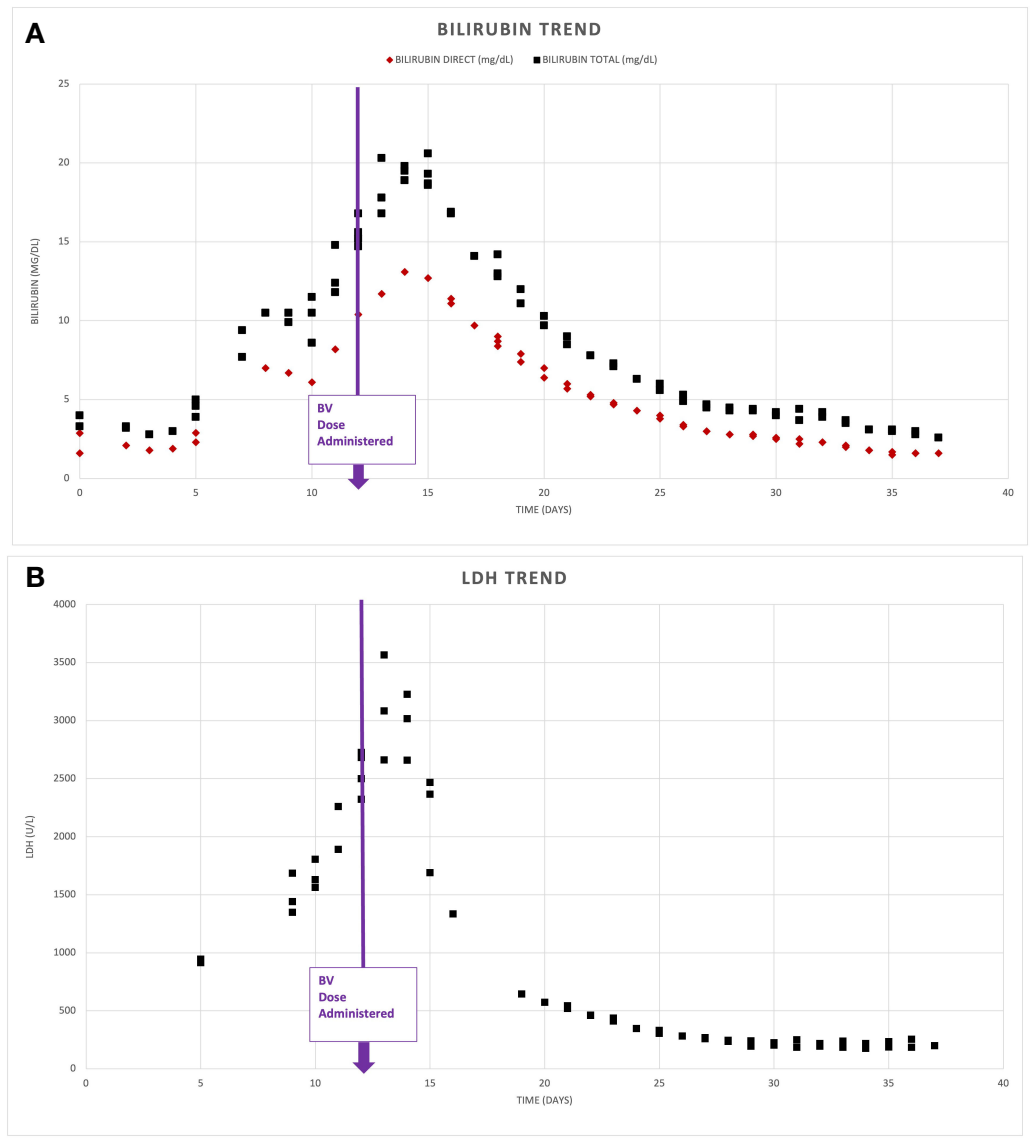
Serum biomarker response. Depicts serum bilirubin levels **(A)** and lactate dehydrogenase (LDH) **(B)** noted on presentation and following administration of Brentuximab Vedotin (BV) on day 12 of admission.

In view of the mixed hepatocellular and cholestatic liver impairment, with total bilirubin peaking at > 20 mg/dL, most cytotoxic therapies were relatively contraindicated; therefore, monotherapy with dose reduced BV at 1.2 mg/kg q21 days for 2 cycles was initiated, with marked improvement in liver enzymes and bilirubin. DA-EP_CH (dose-adjusted etoposide, prednisone, cyclophosphamide, doxorubicin), with dose modification for hyperbilirubinemia and omission of vincristine, were added to therapy 3 days following cycle 2 of BV, once bilirubin was less than 3 mg/dL. A repeat PET-CT revealed near complete resolution of abnormal FDG uptake and lymphadenopathy, with only residual mild lymphadenopathy in the left axilla and right inguinal regions, Deauville 2 (**Figure 2**). There was also a marked decrease in previously seen hepatosplenomegaly. The patient completed five additional cycles of DA-EP_CH and full-dose BV at 1.8 mg/kg. The course of laboratory findings, including serum bilirubin and trends are depicted in **Figure 3**. Post-treatment PET-CT revealed resolution of FDG-avid adenopathy, Deauville 1, but new intense uptake in the gallbladder (**Figure 2**). Ultrasonography revealed several foci of adenomyomatosis in the gallbladder and mildly prominent, echogenic liver consistent with steatosis. Repeat PET-CT 6 months later showed no evidence of FDG-avid lymphadenopathy, Deauville 1. On clinical follow-up 2 years after initial therapy, the patient remained without evidence of recurrent disease and had returned to full-time work.

## Discussion

GZL often presents a diagnostic dilemma, requiring adequate biopsy specimens and a high index of suspicion to lead to an accurate diagnosis as is seen in our case. To optimize diagnostic yield, excisional samples are preferred, with high tumor cell content and associated stromal background (3). Histopathologically and immunophenotypically the disease shares features of cHL and primary DLBCL, often with strong expression of both CD20 and CD30, and expression of MUM1. Additionally, PAX5 and OCT2 positivity are observed in most cases, with intermediate rates of CD15 expression. EBV is almost universally not detected (2). Importantly, GZL cases should lack the typical architectural features of cHL such as nodular growth and fibrosis. Our case demonstrated CD15 and CD30 positivity on both the initial and subsequent core biopsies but lacked morphologic criteria consistent with cHL, namely absence of a typical inflammatory background. The axillary lymph node specimen demonstrated sheets of large pleomorphic atypical lymphocytes with scattered interspersed small T-lymphocytes. B-cell origin of the atypical lymphocytes was established based on labelling for OCT-2, BOB1, and relatively strong expression of PAX-5, as CD20 expression was absent (**Figure 1**).

Clinically, cases often present with mediastinal disease, as seen in cHL and primary mediastinal large B-cell lymphoma (PMBCL), but increasingly, non-mediastinal cases are being identified (9). Primary mediastinal disease in GZL has been associated with younger age of presentation versus non-mediastinal disease (median age 29-35 versus 51-55) per several case series (3, 8). Our case demonstrates non-mediastinal disease characterized by lymphadenopathy and extra-nodal involvement of the spleen and bone marrow. Male gender is most common, with a male/female ratio of ~2:1 seen in most series (8). Hypoalbuminemia at diagnosis (<4.0 g/dL) as seen in our case has been correlated with markedly worse outcomes (PFS HR 5.06, CI95 1.11-23.19, p = .03) (3).

The incorporation of non-mediastinal gray-zone lymphoma cases into the gray-zone lymphoma category has encountered challenges, especially with the growing recognition of molecular and genetic similarities to DLBCL NOS. In light of these findings, the recent revision of lymphoma classification by WHO in 2022 has redefined cases like ours as DLBCL NOS (10).

The molecular pathogenesis and mutational landscape of GZL remain poorly characterized, given the paucity of cases. Comparative gene expression analysis by Pittaluga et al. examined gene expression profiles in 103 genes from 20 mediastinal gray zone lymphoma (MGZL), 18 cHL and 17 PMBCL cases, and found MGZL to have distinctive clustering, but, with biologic features more similar to cHL than PMBCL (11). The gene expression patterns in MGZL and cHL indicate reduced B-cell differentiation compared to PMBCL:They exhibit lower expression of germinal center B-cell (GCB) and Interferon regulatory factor 4 (IRF4) genes. Additionally, there is an increase in gene signatures related to infiltrating T-cells and macrophages, including IL6ST, CTLA4, CD28, ICOS, IL1R2, IL32, and IL7R.9 (9).

Another larger GZL characterization by Sarkozy et al. evaluated 50 GZL cases with exome sequencing or targeted panel testing (217 genes) (12). Almost all cases (49/50) displayed a coding mutation in at least one of the genes tested in the panel. The most recurrently mutated gene was *SOCS1* (40%) suggesting that increased JAK/STAT signaling may contribute to the pathogenesis of GZL. Another 29% of cases demonstrated abnormal NF-KB signaling (*NFKBIE* mutation) and *TP53* mutation was noted in 18% of cases (10).

**Figure 4:**
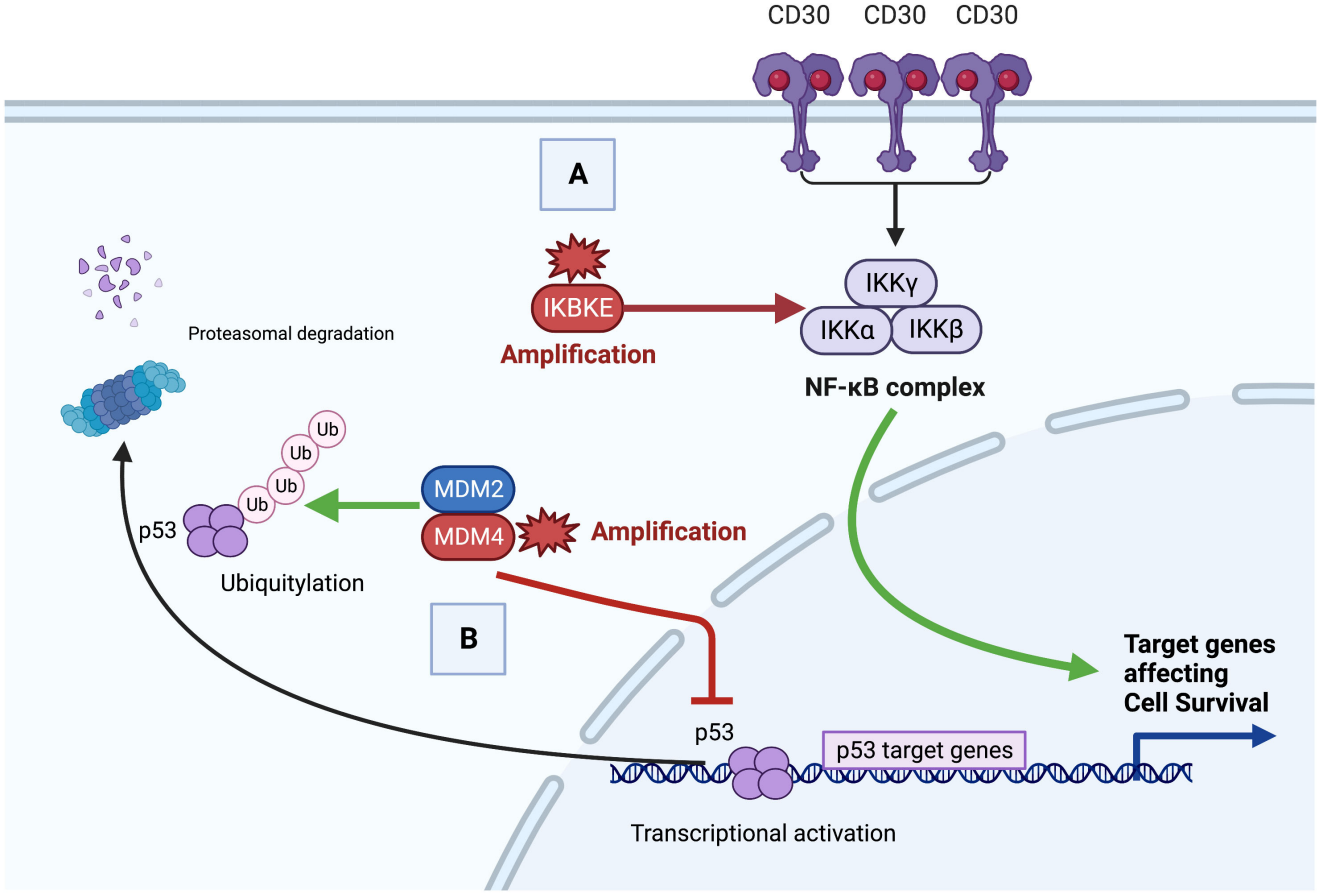
Proposed oncogenesis of gray zone lymphoma with IKBKE and MDM4 alteration. **(A)**. Constitutive Nuclear factor kappa B (NF-κB) activation by canonical and noncanonical signaling. Aberrant canonical activation of NF-κB signaling can occur via increased cell surface receptor activation (e.g. CD30) leading to phosphorylation of the IKK complex. Non-canonical activation can occur via direct phosphorylation by IKBKE. Increased NF-κB activity promotes anti-apoptotic and pro-proliferative target genes leading to oncogenesis. **(B)** Loss of p53 tumor suppressor. The MDM2/MDM4 dimer targets p53 for Ubiquitylation and p53 proteasomal degradation. Overexpressed or amplified MDM4 inhibits p53 target gene transcription in the nucleus and targets p53 protein for proteasomal degradation. Decreased p53 tumor suppressor leads to loss of malignant cell apoptotic regulation.

Next generation sequencing in our case demonstrated amplification of *IKBKE* (11 copies) and *MDM4* (11 copies) and mutation of *ASXL1* (D740fs*6). A summary of the potential role of these genes in the pathobiology of this GZL case is depicted in **Figure 4**.

IKBKE is a member of the inducible I-Kappa Beta kinase complex family, which includes IKKα, IKKβ, and IKKγ (13). Extracellular stimuli such as pro-inflammatory cytokines IL-1, IL-6, TNFα, and bacterial lipopolysaccharides (LPS), as well as B or T-cell activation (via cell surface receptors such as CD30) can lead to canonical activation of NFKB signaling via phosphorylation of the IKB complex, which leads to poly-ubiquination, degradation and subsequent release of NF-KB dimers for translocation to the nucleus (14). Canonical NF-KB is regulated by auto-regulatory feedback mechanisms. IKBKE phosphorylates the complex in a non-canonical manner, as such over-amplification or expression, can lead to increased NF-KB activity and subsequent anti-apoptotic and pro-proliferation cellular mechanisms. Likewise, IKBKE is highly expressed in malignant solid tumors including 30% of breast carcinomas, 88% of glial tumors, 90% of hepatocellular carcinoma and others (15, 16).

Aberrant NF-κB signaling has been implicated in aggressive lymphoid malignancies including cHL, PMBCL and activated B-cell (ABC) subtype DLBCL (17). Similarly, gene-expression profiling in MGZL cases demonstrate high NF-κB signatures with non-canonical expression (9). Large-scale DNA methylation analysis of micro-dissected GZL tumor cells has also suggested a central role for NF-κB pathway activation in the pathogenesis of GZL (18). In cHL, CD30 expression and EBV related LMP expression lead to an alternative pathway of NFKB activation (12). Small molecule inhibitors targeting the IKB complex are thus of great interest as potential targets in the treatment of lymphoid and other malignancies (19).

Murine double minute 2 (MDM2) and Murine double minute 4 (MDM4) are potent negative regulators of the p53 tumor suppressor protein (20). The MDM2/MDM4 dimer polyubiquitinates p53, targeting it for proteasomal degradation, and inhibits p53 transcriptional activity (17). MDM4 amplification and overexpression is seen in hematologic malignancies including acute myeloid leukemia, anaplastic large cell lymphoma and Burkitt lymphoma (21, 22). MDM4 knockdown leads to p53 activation and tumor regressions (17), prompting ongoing studies of MDM4 inhibitors (17, 23).

The optimal first line treatment approach in GZL is unknown due to lack of prospective data. Case series support the use of B-cell targeted therapy with R-CHOP or R-DA-EPOCH rather than cHL regimens such as ABVD +/- R despite gene expression profiling suggesting biological similarities to cHL over PMBCL (4, 9). Our patient presented with end-organ impairment (elevated transaminases and direct hyperbilirubinemia) limiting the use of cytotoxic chemotherapies.

BV is an antibody-drug conjugate which combines anti-CD30 monoclonal antibody with a microtubule-disrupting agent. The manufacturer recommends dose reduction to 1.2 mg/kg with mild hepatic impairment (Child-Pugh A) and to avoid use in moderate (Child-Pugh B) and severe (Child-Pugh C) hepatic impairment (7). Anti-CD30 therapy with dose-reduced BV (1.2 mg/kg) has previously been shown to be a safe bridging therapy for cHL patients with liver impairment (6). CD20 directed therapy with Rituximab was also a consideration, however, in view of the absence of labeling for CD20 by immunohistochemistry in the second biopsy, the benefit of this therapy appeared less clear. After 2 cycles of dose-reduced BV our patient achieved normalization of organ function and was able to receive full-dose BV-CHP and a durable complete remission. The frequency of CD30 expression in GZL, and our understanding of GZL pathobiology, suggest CD30 mediated NF-κB signaling is oncogenic, thus targeted treatment with BV may improve outcomes. Further utilization of BV-CHP as first-line treatment of GZL in larger trials would be valuable towards addressing this unmet need.

## Data availability statement

The original contributions presented in the study are included in the article/supplementary material. Further inquiries can be directed to the corresponding author.

## Ethics statement

Written informed consent was obtained from the individual(s) for the publication of any potentially identifiable images or data included in this article.

## Author contributions

RA: Data curation, Supervision, Validation, Visualization, Writing – original draft, Writing – review & editing. TC: Visualization, Writing – review & editing. BP: Data curation, Visualization, Writing – original draft, Writing – review & editing. TA: Writing – review & editing. JB: Methodology, Supervision, Writing – review & editing.
